# Morphological Phenotyping of Organotropic Brain- and Bone-Seeking Triple Negative Metastatic Breast Tumor Cells

**DOI:** 10.3389/fcell.2022.790410

**Published:** 2022-02-17

**Authors:** Ariana Joy L. DeCastro, Marina A. Pranda, Kelsey M. Gray, John Merlo-Coyne, Nathaniel Girma, Madelyn Hurwitz, Yuji Zhang, Kimberly M. Stroka

**Affiliations:** ^1^ Fischell Department of Bioengineering, University of Maryland, College Park, MD, United States; ^2^ Department of Biology, University of Maryland, College Park, MD, United States; ^3^ Department of Epidemiology and Public Health, University of Maryland, Baltimore, MD, United States; ^4^ Marlene and Stewart Greenebaum Comprehensive Cancer Center, University of Maryland, Baltimore, MD, United States; ^5^ Biophysics Program, University of Maryland, College Park, MD, United States; ^6^ Center for Stem Cell Biology and Regenerative Medicine, University of Maryland, Baltimore, MD, United States

**Keywords:** cell migration, cell morphology, tumor cells, cell mechanical characterization, focal adhesions

## Abstract

Triple negative breast cancer (TNBC) follows a non-random pattern of metastasis to the bone and brain tissue. Prior work has found that brain-seeking breast tumor cells display altered proteomic profiles, leading to alterations in pathways related to cell signaling, cell cycle, metabolism, and extracellular matrix remodeling. Given the unique microenvironmental characteristics of brain and bone tissue, we hypothesized that brain- or bone-seeking TNBC cells may have altered morphologic or migratory phenotypes from each other, or from the parental TNBC cells, as a function of the biochemical or mechanical microenvironment. In this study, we utilized TNBC cells (MDA-MB-231) that were conditioned to metastasize solely to brain (MDA-BR) or bone (MDA-BO) tissue. We quantified characteristics such as cell morphology, migration, and stiffness in response to cues that partially mimic their final metastatic niche. We have shown that MDA-BO cells have a distinct protrusive morphology not found in MDA-P or MDA-BR. Further, MDA-BO cells migrate over a larger area when on a collagen I (abundant in bone tissue) substrate when compared to fibronectin (abundant in brain tissue). However, migration in highly confined environments was similar across the cell types. Modest differences were found in the stiffness of MDA-BR and MDA-BO cells plated on collagen I vs. fibronectin-coated surfaces. Lastly, MDA-BO cells were found to have larger focal adhesion area and density in comparison with the other two cell types. These results initiate a quantitative profile of mechanobiological phenotypes in TNBC, with future impacts aiming to help predict metastatic propensities to organ-specific sites in a clinical setting.

## Introduction

Breast cancer organotropic metastasis is the non-random pattern of breast cancer cells metastasizing to organs such as the bone, lung, liver, and brain (Gao et al., 2019). The “seed and soil theory” hypothesizes that the cancer cells (seeds) have a favorable relationship with certain environments (soil) (Fokas et al., 2007). In support of this theory, a study done in 886 breast cancer patients showed that 38.9% of patients with triple negative breast cancer (TNBC) developed metastases to the bone, while 25.2% of TNBC patients developed metastases to the central nervous system (Bartmann et al., 2017). Further, a systematic literature review of 96 studies in the EMBASE and MEDLINE data bases found that TNBC was associated with a shorter time to brain metastasis compared to luminal subtypes (hormone receptor positive) (Koniali et al., 2020).

The gold standard for treating TNBC is neoadjuvant therapy, which includes chemotherapy prior to invasive surgery, though there is still a high chance of relapse within 5 years (Medina et al., 2020). This disease has a higher incidence rate in younger (Partridge et al., 2016) and African American women (Scott et al., 2019). Unfortunately, African Americans are less likely to be diagnosed with localized cancer, a phase which is easier and more successfully treated (Lisovicz et al., 2008). There is a critical need to diagnose this subtype of breast cancer at an earlier rate, especially for the African American community, in order to increase chances of early, and more successful treatment. It is possible that a better understanding of the morphological and biomechanical cell phenotypes associated with organotropic TNBC will lead to improved future treatments and reduction of this health disparity.

One factor that may contribute to organotropic TNBC metastasis is the tumor cell–microenvironment interactions. MDA-MB-231 brain-seeking cells have been shown to interact with astrocytes at the blood-brain barrier, leading to activation of the immune response which contributed to chemoresistance and tumor growth (Chen et al., 2016). Further, we have shown that secreted factors from astrocytes alter MDA-MB-231 morphology and 2D migration (Shumakovich et al., 2017). Other studies have explored MDA-MB-231 behavior in bone-related microenvironments. Blocking CXCL12, a chemokine involved in hematopoietic stem cell homing to bone marrow, reduced MDA-MB-231 migratory behavior, and metastasis to the lung and lymph-node. Therefore, there is speculation that CXCL12 chemokine could be involved in breast cancer cell homing to the bone (Müller et al., 2001). Bone-seeking MDA-MB-231 cells have also been shown to overexpress parathyroid hormone protein (PTH-rP) which regulates bone remodeling (Hayman et al., 1991; Yin et al., 1999).

In addition to understanding the mechanisms behind organotropic metastasis, it is equally important to be able to identify unique characteristics of organotropic breast cancer cells that could distinguish them in a heterogeneous tumor cell population. An unanswered question is whether or not organotropic cells can be identified (or distinguished) by their physical characteristics, such as morphology, migration, and mechanical properties. One study isolated single cell clones of the parental MDA-MB-231 cell line, determined that some of these clonal lines had a unique cell morphology, and showed that certain clones were able to establish large secondary tumors in the lungs while others were only slightly tumorigenic; the degree of tumorigenicity and metastatic capability was similar among single cell clones with the same morphology (Wu et al., 2020). A similar study has been done in pancreatic cancer cells and showed that cells with morphological heterogeneity were more prevalent in secondary lung tumors, compared to primary tumor cells (Wu et al., 2015). Identifying the unique physical characteristics of organotropic tumor cells may have potential application in diagnosing metastatic risk of cancer patients and long-term novel therapeutic developments to reduce cancer disease progression. In this study, we used TNBC cell lines (MDA-MB-231) that have been conditioned to metastasize preferentially to brain or bone tissue (Yoneda et al., 2001), with a goal of building a profile of phenotypic characteristics that could distinguish these subtypes in a heterogeneous tumor cell population.

Brain and bone tissue have distinct microenvironments. According to the seed and soil theory, components of the microenvironment such as extracellular matrix composition, and mechanical cues may play a role in organotropic metastasis. To form secondary tumors in brain tissue, the metastasizing cancer cells may take a route across the blood brain barrier (BBB), which is composed of brain endothelial cells and supported by astrocytes and pericytes, which help maintain homeostasis and regulate transport into the brain perivascular space (Abbott and Yusof, 2010). Breast cancer cells also must navigate the basement membrane of brain microvasculature, which is mainly composed of collagen IV, laminin, fibronectin, and other proteins. Furthermore, brain tissue contains non-fibrillar collagens types IV and VI, as well as hyaluronic acid and other proteins (Ananthanarayanan et al., 2011; Lau et al., 2013; George and Geller, 2018), and is on the order of ∼1 kPa in stiffness (Rao et al., 2013; Antonovaite et al., 2018). Collagen I does not contribute to the brain tissue mechanics due to its low concentration (Linka et al., 2021). On the other hand, the bone microenvironment is composed of an extracellular matrix mainly composed of collagen I, which enhances differentiation, adhesion, proliferation, and migration (Schor, 1980; Schor et al., 1980). Collagen I is interspaced with inorganic hydroxyapatite (Alford et al., 2015), other noncollagenous proteins, cells including osteoblasts and osteoclasts that regulate bone remodeling (Brook et al., 2018), and a vascular network with endothelial cells lining the lumen surface (Watson and Adams, 2018). Tumor cells in this environment would have to navigate a stiffer matrix, as mature bone tissue has a Young’s and shear modulus in the GPa range (Mulder et al., 2008) and bone marrow tissue has a stiffness range of 0.3–25 kPa (Barney et al., 2016). The differing mechanical properties and protein composition of brain versus bone motivate questions about whether brain-seeking and bone-seeking breast tumor cells display different functionally-relevant behaviors, such as morphology and migration, in brain versus bone tissue.

Previous studies have already explored some distinguishing characteristics of the MDA-MB-231 brain-seeking (MDA-BR) and bone-seeking (MDA-BO) clones. A proteomic study found that the MDA-BR clones displayed alterations in pathways related to cell signaling, cell cycle, metabolism and extracellular matrix remodeling compared to the parental MDA-MB-231 (MDA-P) clone (Dun et al., 2015). Yoneda *et al.* showed that MDA-BO cells have a higher production (compared to MDA-P and MDA-BR cells) of the hormone PTH-rP, which plays a role in the development of bone metastases (Yoneda et al., 2001). MDA-BR cells also have higher expression of matrix metalloproteinases (MMPs) 1 and 9 mRNA levels compared to the MDA-P and MDA-BO cells (Stark et al., 2007). Furthermore, one study showed that cell adhesion on brain- and bone-like extracellular matrices may be able to distinguish the organ-seeking clone subpopulations and predict metastatic risk (Barney et al., 2015). These previous studies have served as the motivation to further explore cell phenotypic characteristics that could help to identify organ-seeking clones within heterogeneous tumor populations. Understanding these phenotypes could motivate the development of novel treatments to target tumor cell subsets and their preferred microenvironment as well as a method of diagnosing metastatic risk to specific organs in a clinical setting.

Here, we explored whether cell phenotypic characteristics such as morphology, cell migration, and mechanical properties may differ between organ-seeking clones. We show that MDA-BO clones have a smaller and more protrusive morphology that may be influenced by successive *in vitro* passaging. Further, the MDA-BO clone has an altered chemokinetic migratory response on two-dimensional collagen I-coated substrates in comparison with MDA-BR and MDA-P cells. Finally, both the mechanical properties (i.e., Young’s modulus) and cell morphological properties varied between the MDA-P, MDA-BO, and MDA-BR clones as a function of substrate stiffness. These unique clonal phenotypes provide further insights into the functional differences between brain- and bone-seeking metastatic tumor cells.

## Materials and Methods

### Cell Culture

MDA-MB-231 parental (MDA-P), brain-seeking (MDA-BR), and bone-seeking (MDA-BO) clones were provided by Dr. Toshiyuki Yoneda in Osaka, Japan. Organ-seeking clones were developed in his lab using a protocol described previously (Yoneda et al., 2001). Briefly, MDA-MB-231 cells were injected into mice and allowed to metastasize to the brain or bone. This process was repeated until a population that solely metastasizes to either the brain or the bone was established. Upon arriving in our lab, the cells were checked for authenticity using STR testing (Laragen, Inc.), and all three cell lines best matched the MDA-MB-231 line (see Supplementary Material for full results provided by Laragen, Inc.). The cells were cultured in medium consisting of Dulbecco’s Modified Eagle’s Medium (DMEM) supplemented with high glucose (ThermoFisher Scientific, Waltham, MA, United States), 10% Fetal Bovine Serum (FBS; HyClone Characterized GE Healthcare, Pittsburgh, PA, United States or ThermoFisher Scientific), and 1% Penicillin-Streptomycin 10,000 U/ml. Cells were washed with Phosphate-Buffered Saline (PBS) (VWR, Radnor, PA, United States), and detached with 0.25% Trypsin-EDTA (ThermoFisher Scientific). Cells were cultured at 37°C, 50% humidity, and 5% CO_2_:95% air. Cells were passaged every 2 days (up to passage 12) when about 80% confluency was reached. Unless otherwise noted, trials from different passages were pooled, while Figure 2 specifically assessed passage-dependence.

### Phase Contrast Imaging and Measurement of Suspended and Adherent Cell Morphology

To assess the morphology of suspended (non-adherent) cells, a total of 4 × 10^5^ cells were plated into 6-well non-tissue culture treated plates (VWR) and imaged immediately. Ten-micron diameter (9.94 ± 1.01 µm according to manufacturer for specific batch) Envy Green fluorescent beads (Bangs Laboratories, Inc., Fishers, IN, United States) were suspended in blank wells of the same plates to validate imaging and analysis based on the known diameter. For cells in suspension (and beads), images were modified in ImageJ for maximum brightness and contrast to achieve the sharpest contours. The image was then converted to binary black and white after thresholding. The ImageJ function to fill holes was applied and the ImageJ built in particle analyzer was used to analyze particles 300–1,250 pixels^2^ in area with circularity values of 0.65–1 to eliminate any aggregates from analysis. To assess the morphology of adherent cells, 1 × 10^5^ cells were plated into 6-well tissue culture treated plates (VWR) and allowed to attach overnight. Phase contrast imaging was performed using a 20x objective. ImageJ was used to process images and quantify area, inverse aspect ratio, circularity, and solidity. Inverse aspect ratio is defined as the ratio of the minor axis to the major axis. Solidity is defined as the ratio of the area to the convex area. Circularity is the ratio of 4πA to *P* ^*2*^, where *A* is the projected area of the cell in the image, and *p* is the perimeter of the cell.

### 2D Migration Assays

For 2D cell migration experiments, cells were plated onto substrates coated with collagen I from rat tail (Sigma Aldrich, St. Louis), fibronectin from human plasma (Sigma Aldrich), or poly-d-lysine hydrobromide (PDL) (Sigma Aldrich). Collagen I and fibronectin were dissolved in PBS, and PDL was dissolved in sterile milliQ water. Twenty-four well glass bottom plates (MatTek, Ashland, MA) were coated with 300 µl (per well) of 20 μg/ml of the varying protein solutions and incubated for 1 h at 37°C. Following incubation, wells with collagen and fibronectin were washed 3 times with PBS, while wells with PDL were washed 3 times with sterile milliQ water. After washing the substrates, 1 × 10^4^ cells were plated into each well. Cells were imaged and analyzed as described below.

### Microchannel Device Fabrication and Preparation

Microchannel devices were fabricated as we previously described in detail (Balzer et al., 2012; Doolin and Stroka, 2018). Once fabricated, the polydimethylsiloxane (PDMS) devices were sonicated in 100% ethanol (Pharmco) for 5 min. The PDMS devices and 35 mm by 75 mm glass coverslips were rinsed with deionized water, followed with 100% ethanol, and then dried thoroughly with pressurized air. Both the PDMS devices and coverslips were plasma-treated using a plasma cleaner (Harrick Plasma, Ithaca, NY, United States) and pressed together for irreversible bonding. 40 μl of type I rat tail collagen (20 μg/ml) solution was pipetted into each inlet and outlet of the device, the device was incubated for 1 h at 37°C, and then the device was washed two times by pipetting PBS into and out of all wells of the device. Meanwhile, cells growing in culture were washed with PBS, detached with 0.25% Trypsin-EDTA (ThermoFisher Scientific), centrifuged, and resuspended to a final concentration of 1 × 10^5^ cells/25 µl. Twenty-five microliters of cell suspension were added to the cell inlet, and the device was incubated for 5 min at 37°C for initial cell seeding. Excess liquid was removed from the cell inlet and outlet, and 50 µl of serum-free media was added to the bottom inlet, along with the 2 lower inlets of the upper channel. Fifty microliters of serum-full media were added to the top-most inlet of the main upper channel.

### Polyacrylamide Gel Preparation

Polyacrylamide (PA) gels (∼80 μm thick) were formed using a method initially described by Wang and Pelham (Wang and Pelham, 1998) and described in our previous publications (Stroka and Aranda-Espinoza, 2009; Stroka and Aranda-Espinoza, 2011; Stroka et al., 2012; Hamilla et al., 2014; Gray et al., 2019) on 22 × 22 mm coverslips (ThermoFisher Scientific). PA gels were coated with 50 μg/ml collagen I using sulfo-SANPAH activation, also as previously described in our work (Stroka and Aranda-Espinoza, 2009; Stroka and Aranda-Espinoza, 2011; Stroka et al., 2012; Hamilla et al., 2014; Gray et al., 2019). A total of 1 × 10^5^ cells was plated onto each gel. For AFM experiments on gels, coverslips containing gels were secured to 50 mm AFM grade glass bottom dishes (VWR).

### Atomic Force Microscopy

The Young’s modulus of tumor cells on PA gels (as described in the section above) or on glass (50 mm AFM grade glass bottom dishes, VWR) was measured using atomic force microscopy (AFM). After 1 day in culture, AFM was performed on live cells with the stage heated to 37°C. AFM was performed using an Asylum MFP-3D-BIO Atomic Force Microscope with TR400PB(L) probes (Asylum Research) as previously described (Gray et al., 2019). Asylum’s “Get Real” approach was used to measure the spring constant and inverse optical lever sensitivity of TR400PB(L) probes via the Sader method and thermal noise method, respectively. The average spring constant of the cantilevers was within a factor of 1.33 to Asylum’s nominal value of 0.02 N/m and all within the nominal range of 0.01–0.05 N/m. One 100-curve force map covering a 5 μm^2^ area was collected for each cell using a 2 μm force distance, a 1 V trigger point (approximately 1.35 nN), and a scan rate of 0.99 Hz. The Hertz model was used to fit the force curves within Asylum’s Igor Pro-based software using the equation $F=\;\frac{4}{3}*\left(\frac{E}{1-\upsilon^{2}}\right)*\sqrt{r}*\delta^{3/2},$ where *δ* is the measured indentation of the sample and the Young’s modulus *E* was the fitting parameter. The Poisson’s ratio *υ* of the sample was assumed to be 0.45 and the tip radius of curvature *r* was approximately 30 nm. Three biological replicates were performed in which three cells per condition per trial were measured (*n* = 9 cells total, or 900 force curves per condition).

### Cell Migration Tracking and Data Analysis

Images were acquired on an Olympus IX83 microscope (Olympus, Center Valley, PA, United States) using a 10x objective. To maintain the cell viability during imaging, a chamber calibrated to 37°C, 50% humidity, and 5% CO_2_:95% air was used on the microscope stage. Images were taken at 5-min intervals (as specified in figure captions). On the following day, a collection of phase contrast images was taken using a 20x objective. 2D and confined cell migration tracking parameters were determined as described previously (Shumakovich et al., 2017). Cells were tracked over 390–750 min for 2D assays and approximately 720 min for confinement assays. Cells that were migrating out of the frame or dividing were not tracked. Mean squared displacement (MSD) was calculated as previously described (Stroka and Aranda-Espinoza, 2009; Shumakovich et al., 2017). Briefly, the average of the square of the distance traveled was calculated as the MSD between each pair of points. The MSDs were averaged for every time interval and plotted vs. the corresponding time interval. The diffusion coefficient was acquired by fitting MSD vs. time plots to the Langevin-type equation $r^{2}\;=\;4D\left[t-\pi \left(1-e^{-1/\tau }\right)\right]$ , where *D* is the diffusion coefficient, $r^{2}$ is the MSD, *t* is time, and τ is the persistence time.

### Immunofluorescent Staining of Focal Adhesions

Glass-bottom dishes were coated with 20 μg/ml collagen I or a 4:1 ratio of 20 μg/ml collagen IV and 20 μg/ml fibronectin and incubated at 37°C for 1 hour, and then washed at least two times with PBS. A total of 1 × 10^4^ cells were plated into the dishes and incubated overnight. The next day, cells were fixed with 3.7% formaldehyde (Millipore Sigma) for 10 min at room temperature. Following three 5-min PBS washes, the cells were permeabilized with 1% Triton-X 100 (Millipore Sigma) at room temperature. The cells were washed 2 times with PBS for 5 min each. The samples were blocked in 2.5% bovine serum albumin (Millipore Sigma) for 1 h at room temperature. Phospho-Paxillin (Tyr118) antibody (Rabbit, monoclonal antibody, Cell Signaling Technology #2541) or Phospho-FAK (Tyr 397) (Rabbit, monoclonal antibody, and Invitrogen #700255) was diluted with 1% bovine serum albumin at a 1:100 ratio and added to the cells. The primary antibodies were incubated with cells overnight at 4°C and samples were washed twice the next day with PBS for 5 min per wash. The samples were then blocked with 2.5% BSA for 1 h at room temperature. Cells were then incubated with 1:500 phalloidin (Alexa Fluor 488, Life Technologies #A12379), 1:2,500 Hoechst (Hoechst 33,342, trihydrochloride, trihydrate, Life Technologies #H3570, and 1:200 secondary antibody (Alexa Fluor 568 goat anti-rabbit (H + L) #A11011) for 1 h at room temperature. Finally, samples were washed 3 times with PBS for 5 min each and stored at 4°C until imaging.

### Confocal Microscopy and Focal Adhesion Analysis

Fluorescent images were acquired using a FV 3000 RS Olympus Laser Scanning Confocal Microscope (Olympus, Center Valley, PA, United States) using a 60x oil objective. Vertical Z-stack images were taken with the appropriate filters using the FluoView FV31S-SW software. Image brightness was adjusted separately for each channel to optimize visibility, but adjustments were done consistently across image sets. For PY-paxillin and phospho-FAK analysis, ImageJ was used to analyze focal adhesion size and count using images at the cell-substrate interface. Images were converted to 8–bit and background was subtracted at 50 pixels. Auto threshold was determined by the software and the “analyze particles” plug-in was used to determine focal adhesion area and count. A minimum focal adhesion size of 4.14 μm was considered to prevent counting background staining. CellProfiler, a cell image analysis software, and was used to calculate the total cell areas from the actin images. Focal adhesion density was calculated by dividing the number of focal adhesions in a cell image by the total cell area in the corresponding image.

### Statistics

GraphPad (GraphPad, San Diego, CA) was used for all statistical analyses. Normality was tested using the D’Agostino-Pearson normality test (Supplementary Table S1). If the data were found to follow or partially follow (e.g., where some groups in the set were normally distributed, while some were not) a normal distribution, a one-way ANOVA or two-way ANOVA was performed to determine statistical significance. If the data did not follow a normal distribution, a Kruskal Wallis test followed by a Dunn’s multiple comparison was used. Statistical significance was determined by using a cutoff of *p* value less than 0.05. At least three independent trials were conducted for each experiment. Cells from all three experiments were pooled between passages 6–12 for all experiments. A ROUT test was used to identify outliers in the focal adhesion expression data set. The data with outliers excluded were used to perform the statistical analysis as previously described.

## Results

### MDA-BO Cells Have Smaller Area and Diameter in Suspension

**FIGURE 1:**
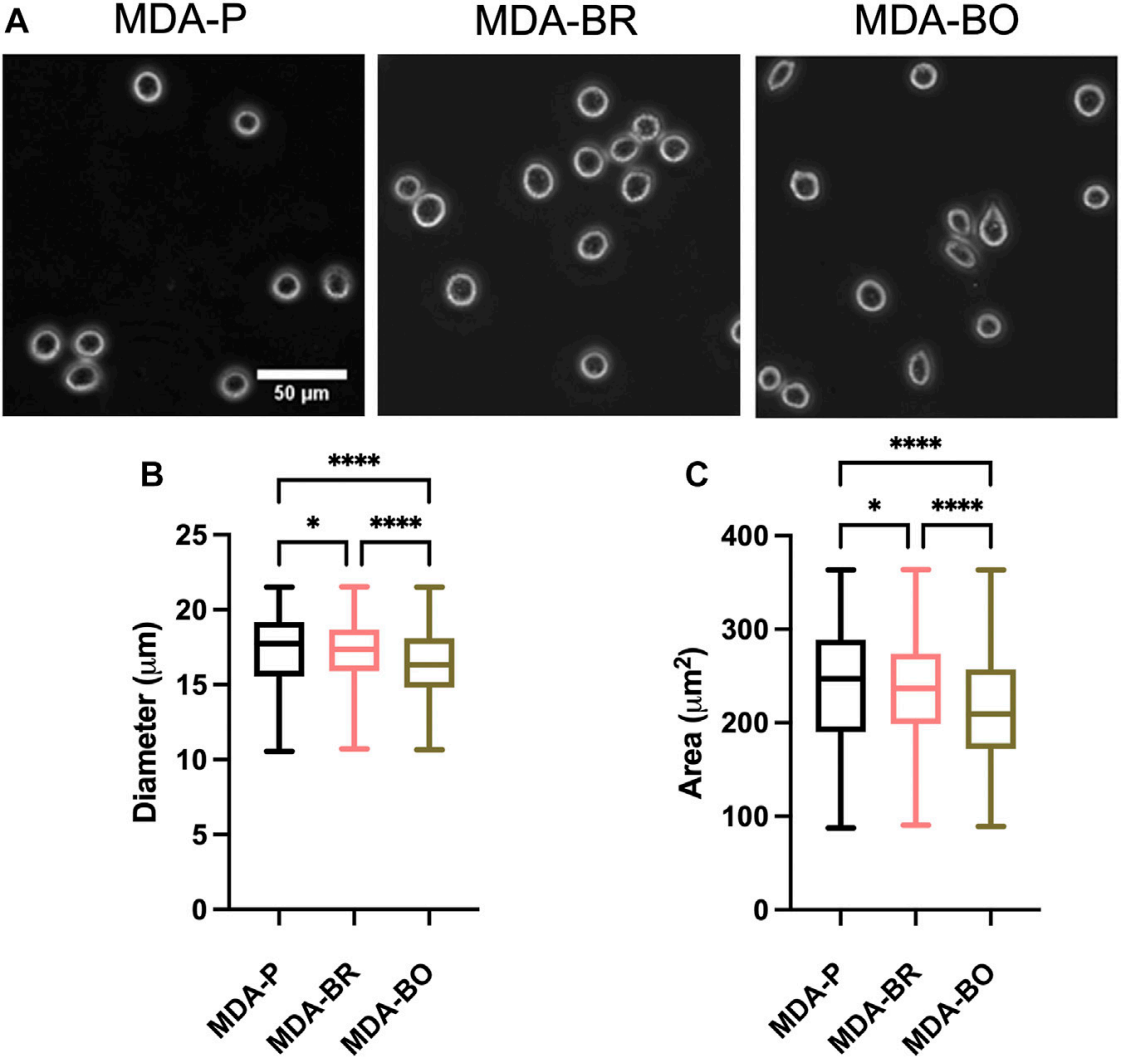
Morphology of suspended parental, brain-seeking, and bone-seeking MDA-MB-231s. **(A)** Phase contrast images of MDA-P, MDA-BR, and MDA-BO cells in suspension. Scale bar represents 50 μm. **(B)** Diameter measured by ImageJ. **(C)** Area of suspended MDA-P, MDA-BR, and MDA-BO cells based on diameter. Box and whisker plots represent the minimum and maximum data points, the median, and the interquartile range. * indicates *p* < 0.05. **** indicates a *p* < 0.0001. Each column of data is from N > 1,277 cells pooled from at least 3 independent experiments. The data in this figure were tested for normality using the D’Agostino and Pearson test and found to be not normally distributed. A Kruskal Wallis test and a Dunn’s multiple comparison test was performed to determine significance.

Breast tumor cells circulate in a suspended state in the blood stream during the metastatic cascade, prior to adhesion to the vascular endothelium (Mentis et al., 2020). Circulating tumor cells are associated with increased risk of metastasis and lower progression-free and overall survival in breast cancer patients (Bidard et al., 2014; Riebensahm et al., 2019; Cristofanilli et al., 2019; Park et al., 2020). We first questioned whether the three breast tumor cell clones had different sizes in suspension. First, to validate morphological analysis of cells in suspension, we added a suspension of 10 µm diameter beads to a glass-bottom dish, imaged the beads using phase contrast microscopy, and used the analyze particles plugin in ImageJ to characterize size of the beads (Figure 1A). Bead diameters using our method were measured to be 11.8 ± 1.25 µm, while bead area was 108.4 ± 16.1 µm^2^. Hence, we moved on to evaluate the size of breast tumor cells in suspension by plating parental and organ-seeking clones into non-treated tissue culture plates and imaging the cells using phase contrast microscopy (Figure 1A). MDA-BO cells were found to have a smaller median diameter (Figure 1B) and area (Figure 1C) when compared to the MDA-BR and MDA-P cells. It is important to note that the cell populations are highly heterogenous in size, even in suspension, as evidenced by the relatively large interquartile ranges of the data (Figures 1B,C). It is possible that differences in cell cycle stage may have contributed to the heterogeneity of suspended cell size, and/or that cells, even in the same cell cycle phase, are innately heterogeneous in size.

### MDA-BO Cells’ Morphology Is Modestly Altered Following Successive *in vitro* Passaging

**FIGURE 2:**
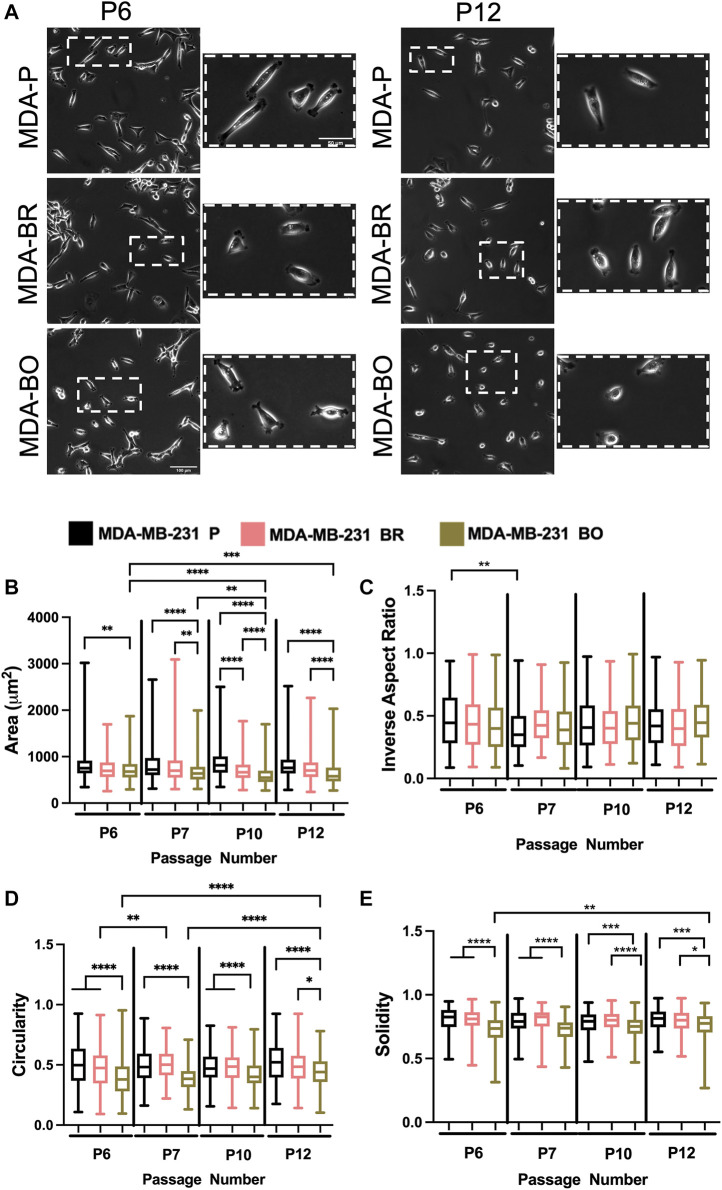
Effects of passage number on cell morphology. **(A)** Phase contrast images of MDA-P, MDA-BR, and MDA-BO cells at Passage 6 (P6) and Passage 12 (P12). Scale bars represent 100 μm (in zoomed out images) or 50 μm (in zoomed insets). Also shown are quantification of cell **(B)** area, **(C)** inverse aspect ratio, **(D)** circularity, and **(E)** solidity as a function of passage number. Box and whisker plots represent the minimum and maximum data points, the median, and the interquartile range. * indicates *p* < 0.05. ** indicates *p* < 0.01. *** indicates *p* < 0.001. **** indicates *p* < 0.0001. Each column of data is from N > 190 cells pooled from at least 3 independent experiments. The data were tested for normality using the D’Agostino and Pearson test. Data for area, inverse aspect ratio, and solidity did not follow a normal distribution, and hence a Kruskal Wallis test and a Dunn’s multiple comparison test were performed to determine significance for those data. Data for circularity partially followed a normal distribution, and hence a two-way ANOVA, and multiple comparison test was performed to determine significance for that data set.

To evaluate the effects of cell passage on the morphology of adherent organ-seeking clones, we plated cells from different passages onto tissue culture treated 6-well plates. Cells were imaged using phase contrast microscopy (Figure 2A), and cell morphological features, including area (Figure 2B), inverse aspect ratio (Figure 2C), circularity (Figure 2D), and solidity (Figure 2E) were analyzed. We found that, for all passages the area, circularity, and solidity of the MDA-BO cells were significantly smaller than the MDA-P cells (Figure 2B). Further, the MDA-BO cells significantly decreased in area, circularity, and solidity (Figures 2B,D,E) between passages 6 and 12. This same trend was not seen in the MDA-P and MDA-BR cells. Inverse aspect ratio (a proxy for cell elongation) was mostly consistent across passages and cell clones (Figure 2C). Overall, these qualitative and quantitative results indicate that the MDA-BO cells are smaller, less circular, and more protrusive than their MDA-P or MDA-BR cell counterparts, and that these morphological features are modestly altered over passage.

### MDA-BO Cells Migrate Over Larger Area During Chemokinesis on Collagen I

**FIGURE 3:**
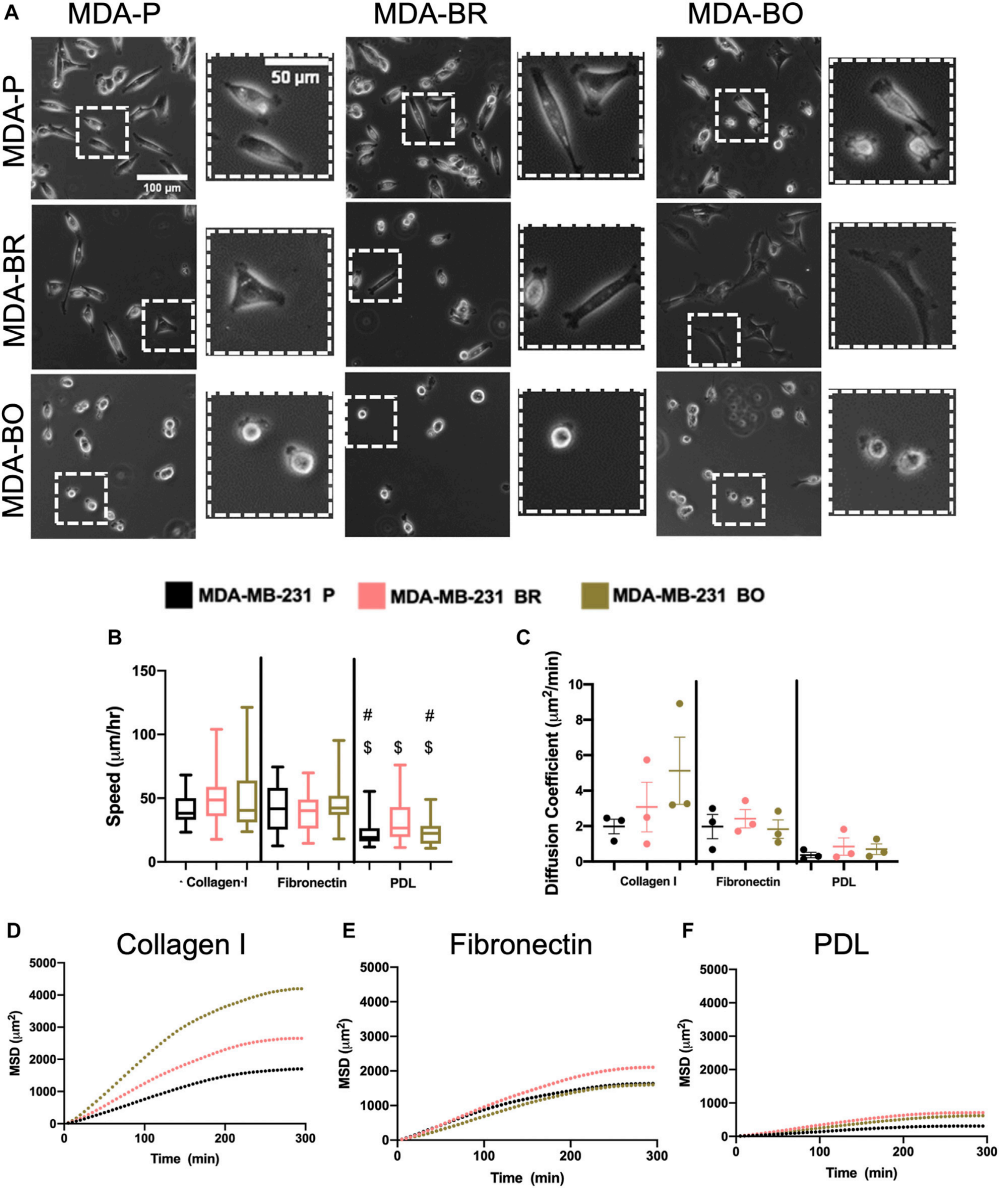
2D migratory characteristics on ECM binding proteins. **(A)** Phase contrast images of MDA-P, MDA-BR, and MDA-BO cells on Collagen I, Fibronectin, or PDL coated substrates. Scale bars represent 100 μm (in zoomed out images) or 50 μm (in zoomed insets). **(B)** Speed tracked for 360 min at 5-min intervals. Groups with # on top are different from corresponding cell type on Fibronectin substrate with *p* < 0.001. Groups with $ on top are different from corresponding cell type on Collagen I substrate with *p* < 0.001. **(C)** Diffusion coefficient calculated from mean squared displacement (MSD) vs. time data (over 300 min), as described in the Methods section. Also shown is mean MSD vs. time interval plots for migration on **(D)** Collagen I, **(E)** Fibronectin, and **(F)** PDL. In panel B, box and whisker plots represent the minimum and maximum data points, the median, and the interquartile range; each column of data is from N > 37 cells pooled from at least 3 independent experiments. Panel B data was tested for normality using the D’Agostino and Pearson test and found to be not normally distributed. A Kruskal Wallis test and a Dunn’s multiple comparison test was performed to determine significance.

Morphological alterations in attached and suspended breast tumor cell clones motivated us to evaluate random migration (i.e., chemokinesis) on three different substrate coatings–collagen I, fibronectin, and poly-d-lysine (PDL). Collagen I is an abundant protein in the bone extracellular matrix (source), while fibronectin is found in the brain basement membrane and extracellular matrix (source); both promote cell integrin-based adhesions. PDL promotes electrostatic interactions between cells and the substrate, thus presumably blocking integrin-based binding. Qualitative inspection of phase contrast images suggested that MDA-BO cells were more protrusive (less solid) on collagen I and on fibronectin (Figure 3A). Meanwhile, during chemokinesis, there were no statistically significant differences in migration speed between cell types on collagen I or fibronectin. On PDL, all cell types were migrating significantly slower compared to their corresponding cell types on collagen I substrate, while only the MDA-P and MDA-BO cells were moving slower compared to a fibronectin substrate (Figure 3B). Meanwhile, on collagen I-coated substrates (but not fibronectin), MDA-BO cells had a higher mean squared displacement (MSD) over time, and diffusion coefficient compared to MDA-BR and MDP-P cells (Figures 3C–F). These results suggest that even though the MDA-BO *speed* was not different from the other clones, the MDA-BO cells covered a larger area over time while migrating, and hence were more *persistent*. Because we observed the largest differences in 2D chemokinetic migration between cell clones on collagen I, the subsequent confined migration, atomic force microscopy, substrate stiffness, and focal adhesion experiments used collagen I as the substrate.

### Confined Migration Speed and Chemotactic Index do Not Vary Between Parental and Organ-Seeking Clones

**FIGURE 4:**
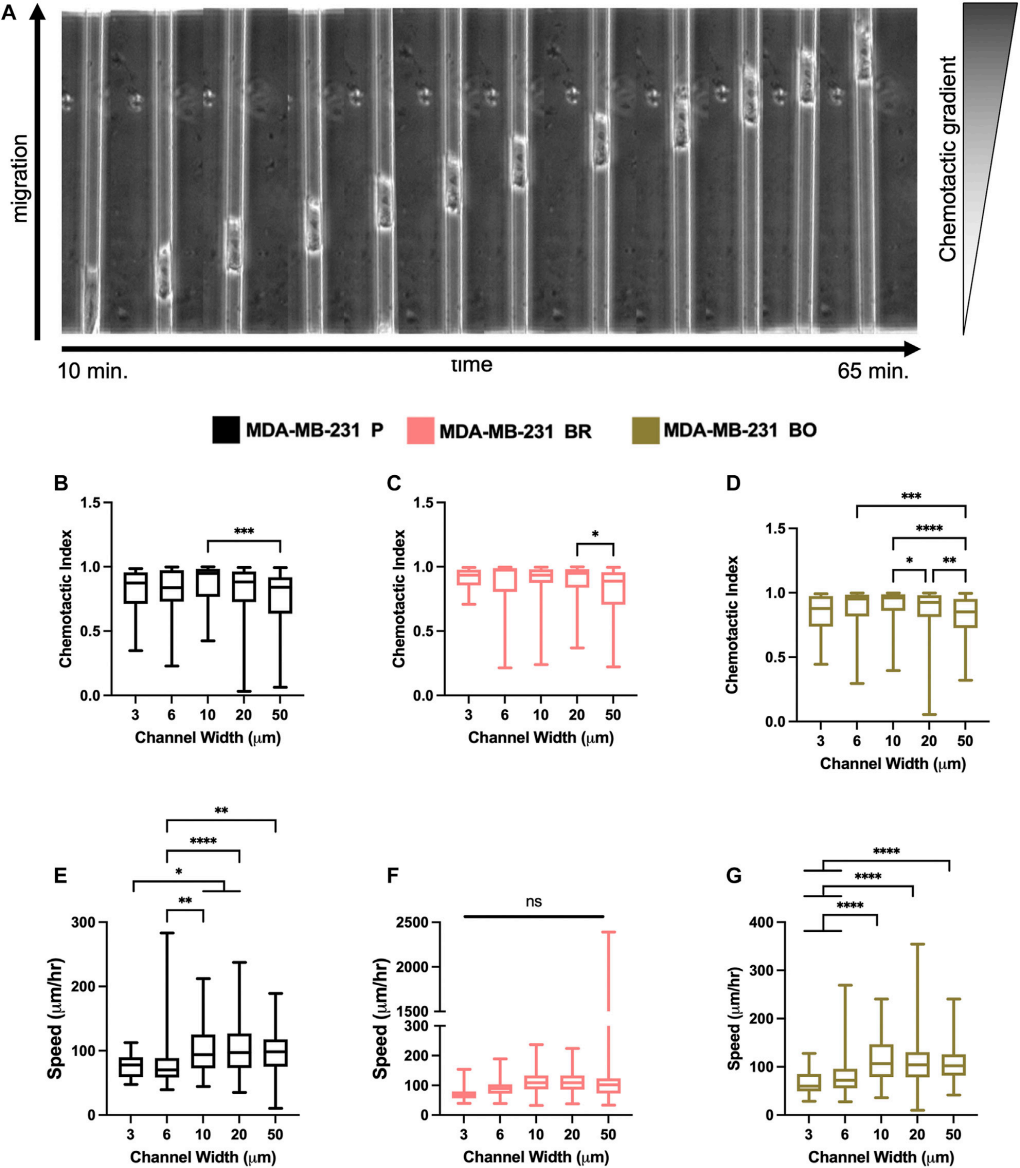
Confined migratory characteristics of organ-seeking clones **(A)** Phase contrast images of an MDA-P cell migrating through a 10 μm channel. Chemotactic indices were quantified for **(B)** MDA-P, **(C)** MDA-BR, and **(D)** MDA-BO for each channel width. Images were taken at 5-min intervals over 12 h. Speed was quantified for **(E)** MDA-P, **(F)** MDA-BR, and **(G)** MDA-BO for each channel width for over 12 h and at 5-min intervals. Box and whisker plots represent the minimum and maximum data points, the median, and the interquartile range. * indicates *p* < 0.05. ** indicates *p* < 0.01. *** indicates *p* < 0.001. **** indicates *p* < 0.0001. Each column of data is from N > 190 cells pooled from at least 3 independent experiments. The data were tested for normality using the D’Agostino and Pearson test and found to be normally distributed. A one-way ANOVA and multiple comparisons test were performed to determine significance these data.

Breast tumor cells experience confinement throughout the metastatic cascade, in migrating through ECMs, during intravasation and extravasation, and while migrating along anatomical features (Balzer et al., 2012; Stroka and Konstantopoulos, 2013). To mimic confinement, microchannel devices with widths varying from 3 to 50 µm were used to evaluate cell migration speed and chemotactic index (CI) in response to a chemotactic gradient (FBS) (Figure 4A). MDA-P and MDA-BO cells generally migrated more persistently (Figures 4B–D) and slower (Figures 4E–G) in highly confined (3, 6, and 10 µm) channels. However, interestingly, that same trend was not observed in the MDA-BR cells, which had similar speeds and persistency across all channel widths.

### Organ-Seeking Clones Have Modest Differences in Stiffness on Soft and Stiff Substrates

**FIGURE 5:**
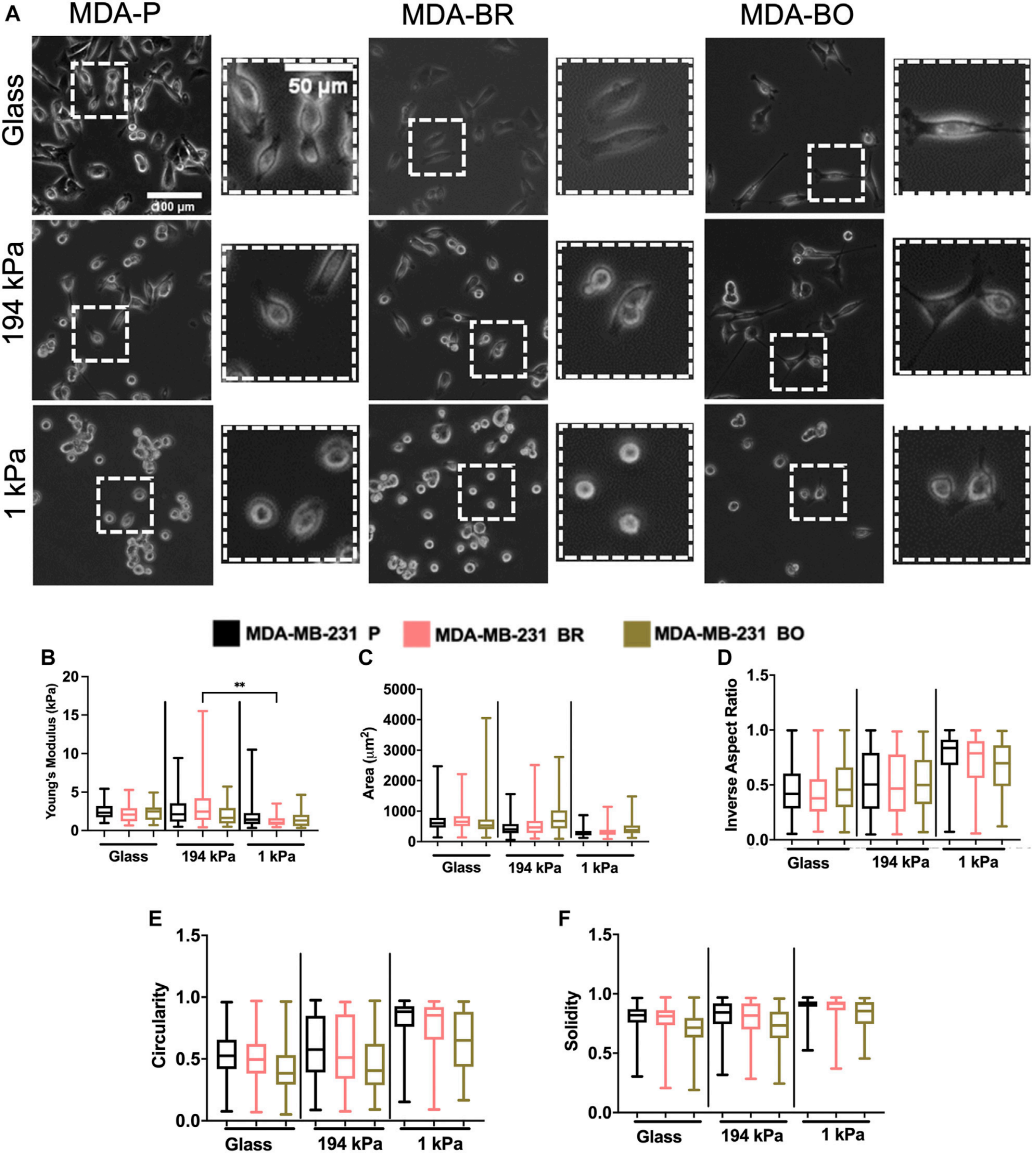
Mechanobiological characteristics of MDA-P, MDA-BR, and MDA-BO cells on stiff and soft substrates **(A)** Phase contrast images of MDA-P, MDA-BR, and MDA-BO cells on glass, 194 kPa, and 1 kPa. Scale bars represent 100 μm (in zoomed out images) or 50 μm (in zoomed insets). **(B)** Young’s modulus from atomic force microscopy of each cell type on glass, 194 kPa, and 1 kPa. Also shown are cell **(C)** area, **(D)** inverse aspect ratio, **(E)** circularity, and **(F)** solidity on each substrate. Box and whisker plots represent the minimum and maximum data points, the median, and the interquartile range. ** indicates *p* < 0.01. Each column of data is from N > 28 cells pooled from at least 3 independent experiments. See additional statistics in Supplementary Table S1. The data were tested for normality using the D’Agostino and Pearson test. Data for Young’s modulus followed a normal distribution for some groups, and hence a two-way ANOVA and multiple comparison test were performed to determine significance for those data. Data for morphology did not follow a normal distribution, and hence a Kruskal Wallis test and Dunn’s multiple comparison test was performed to determine significance for those data sets.

Because the brain and bone-seeking clones target tissues with unique matrix stiffnesses, we hypothesized that one method these organ-seeking cells may use to determine a preferential environment is by sensing the environment’s extracellular matrix stiffness. Further, we wanted to explore whether these clones altered their stiffness in response to the environment, perhaps as a mechanical mechanism that enhances their survival. We compared cells on collagen I-coated glass, which is in the GPa range of stiffness (similar to mature bone tissue), and on collagen I-coated polyacrylamide gels of intermediate stiffness (∼194 kPa) or a stiffness similar to the brain microenvironment (∼1 kPa). The polyacrylamide gels were fabricated, coated with collagen I, and seeded with cells. Atomic force microscopy (AFM) was performed on the cells in force mapping mode and Young’s modulus was calculated by fitting the force vs. distance curves to the Hertz model, as described in the Methods section. MDA-BR and MDA-BO cells’ Young’s moduli on glass were modestly larger in comparison with the respective cell clone on soft 1 kPa gels (Figure 5B). In addition, the MDA-BR cells had a lower stiffness on the 1kPa compared to the 194 kPa (Figure 5B). There were no differences in Young’s modulus between the three cell types for any substrate stiffness.

### MDA-BR and MDA-BO Cells Have a Distinct Morphology Compared to MDA-P Cells on Soft and Stiff Substrates

Since we had previously observed morphological differences between the clones on tissue culture plastic, we quantified whether there were differences in their morphology in response to substrate stiffnesses mimicking either the brain or bone environment. Qualitatively, we observed that all cell types became less spread on soft (1 kPa) substrates (Figure 5A; Supplementary Table S2). We found that the MDA-BO clones had the largest area on both the stiff polyacrylamide gel (∼194 kPa) and soft polyacrylamide gel (∼1 kPa) but the smallest area on glass (Figure 5C) when compared to MDA-P and MDA-BR clones. Further, the MDA-BO clones had the lowest circularity and solidity on all substrates (Figures 5E,F; Supplementary Table S2). Meanwhile, the MDA-BR clones had similar areas to the MDA-P cells on the 1 kPa substrate but had minor differences in the inverse aspect ratio, circularity, and solidity (Figures 5D–F; Supplementary Table S2). Notably, the MDA-BR cells had higher areas compared to the MDA-P cells on both glass and 194 kPa. We note that another study comparing MDA-P and MDA-BR cells has reported a similar trend (Narkhede et al., 2018). Furthermore, both MDA-P and MDA-BR cells had decreasing areas and increasing inverse aspect ratios as stiffness decreased. Finally, all cell types displayed increasing solidity as substrate stiffness decreased.

### MDA–BO Cells Have More Densely Distributed Focal Adhesions on Collagen I Substrate

**FIGURE 6:**
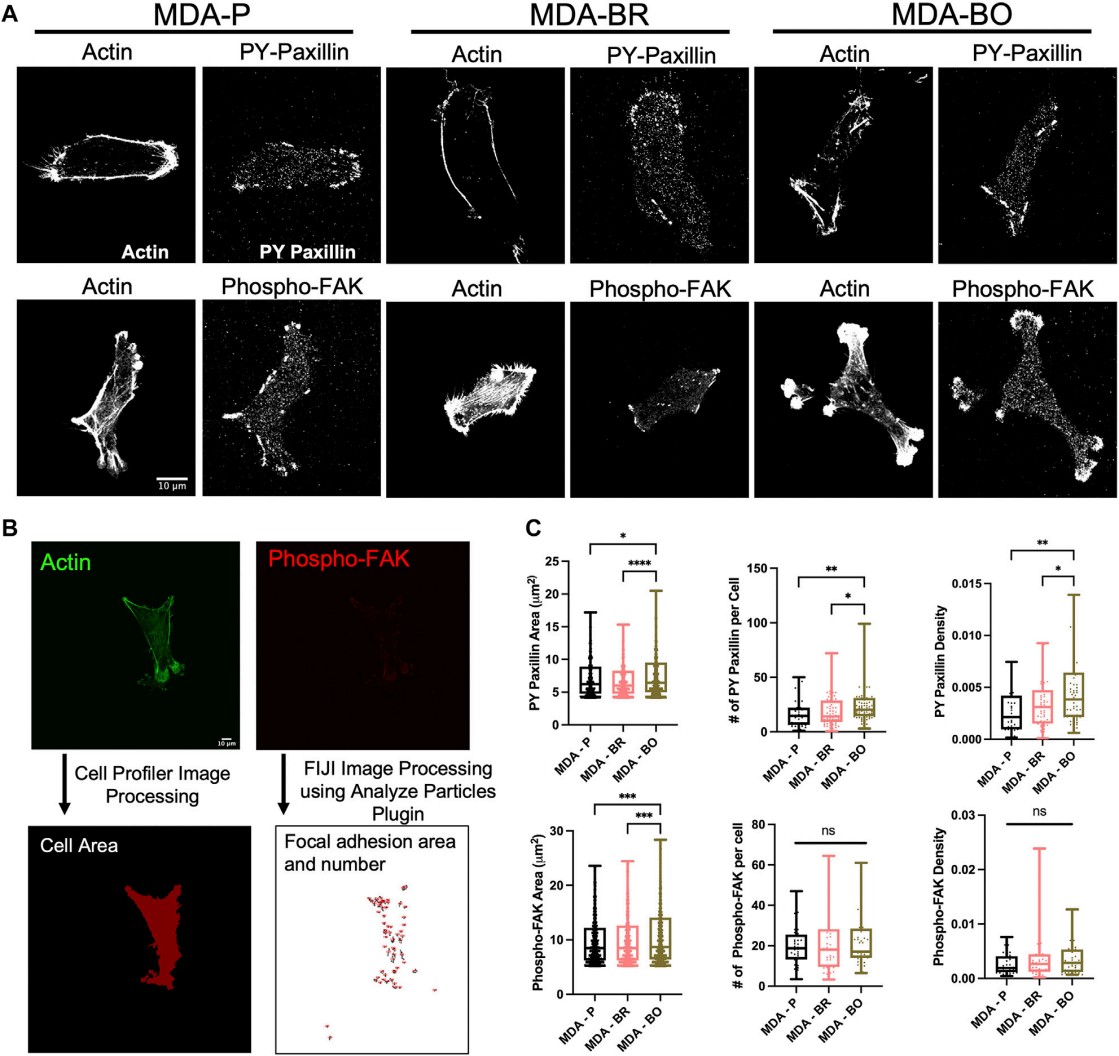
Focal adhesions on Collagen I, and Collagen IV + Fibronectin. **(A)** Confocal images of PY-paxillin and/or Phospho-FAK and Actin on Collagen I or Collagen IV + Fibronectin substrates. Scale bar represents 10 μm. Images are adjusted for best visibility. **(B)** Image processing procedure using FIJI and CellProfiler. Also shown are the area of focal adhesions (FAs), number of FAs per cell, and FA density (number of FAs normalized to cell area) for **(C)** PY-paxillin and Phospho-FAK on Collagen I. Box and whisker plots represent the minimum and maximum data points, the median, and the interquartile range. For FA area, each data point represents the area of an individual FA, and for FA number per cell or FA density, each data point is representative of one cell. * indicates *p* < 0.05. ** indicates *p* < 0.01. *** indicates *p* < 0.001. **** indicates *p* < 0.0001. Each column of data is from N > 53 cells pooled from at least 3 independent experiments. A ROUT test was used to identify outliers in the FA size and density data sets. The data with outliers excluded were used to perform the subsequent statistical analysis. The data was tested for normality using the D’Agostino and Pearson test. Data for number of phospho-FAK FAs per cell and PY-paxillin FA density on collagen I followed a normal distribution for some groups, and hence a one-way ANOVA and multiple comparison’s test were performed to determine significance. For all other measurements, the data were not normally distributed, and hence a Kruskal Wallis test and a Dunn’s multiple comparison test were performed to determine significance.

A previous study has identified a subpopulation of MDA-MB-231s that has a high α5β1 integrin expression, which has been linked to having more prominent focal adhesion expression and higher invasion rates (Mierke et al., 2011). Additional studies have also shown that MDA-BO and MDA-BR cells have higher α5β1 integrin expression (McFarlane et al., 2015; Jahangiri et al., 2017). This motivated us to explore whether there were differences in focal adhesion phenotypes between the MDA-P, MDA-BO, and MDA-PR clones. We imaged PY-paxillin and phospho-FAK, along with F-actin, in cells on collagen I-coated substrates (Figure 6A). We then processed the images using FIJI and Cell Profiler to quantify the area, number of focal adhesions per cell, and density of focal adhesions marked by PY-paxillin or phospho-FAK (Figure 6B). On the collagen I substrate, MDA-BO had a higher PY-paxillin focal adhesion area, count per cell, and density when compared to the other two cell lines (Figure 6C). Furthermore, MDA-BO cells had higher area of phospho-FAK rich focal adhesions compared to the MDA-P & MDA-BR cells, while the focal adhesion count per cell and density were similar (Figure 6C).

## Discussion

Prior studies have shown that larger tumor cells with more protrusions metastasize more and have a greater ability to migrate through blood vessels to distant sites (Stoletov et al., 2010; Yankaskas et al., 2019) suggesting that phenotypic differences between different cell clones may also be indicative of changes in their metastatic potential. Here, our work suggests that MDA-BO cells may be most distinguished from the parental line and MDA-BR clones based on the morphology of suspended and adherent cells, as well as migratory response and PY-paxillin focal adhesion phenotypes on collagen I protein. We speculate that the unique protrusive morphology of the MDA-BO clones may play a role in their ability to establish secondary tumors in the bone tissue. Interestingly, osteocytes, which are the most abundant cell type found in the bone, have long dendritic-like protrusions that radiate into the canaliculi of the bone tissue (Katsimbri, 2017). Hence, the protrusive morphology of the MDA-BO cells may facilitate their migration and/or formation of osteolytic lesions. Meanwhile, the increased PY-paxillin density in MDA-BO cells may enhance cell adhesion and possibly also proliferation within the bone microenvironment. Since the bone microenvironment is rich in collagen I, a higher focal adhesion density on collagen I would be expected for MDA-BO cells, perhaps due to conditioning which led to upregulation of integrin subtypes with different combinations of the α and β subunits that promoted adhesion to collagen I (Seong et al., 2013). Furthermore, MDA-BO cell secretion of PTH-rp hormone (Yoneda et al., 2001), which activates bone resorption (Wein and Kronenberg, 2018), may produce a combinatorial effect, alongside the protrusive morphology and increased focal adhesion density, and to promote bone metastasis. Future work should further explore this combination of phenotypes in identifying risk of bone metastasis in TNBC patients.

Minor differences were found in the adherent and suspended morphology, as well as migration, between the MDA-BR and MDA-P cell line. However, on both glass and 1 kPa soft substrates, the MDA-BR cells showed smaller inverse aspect ratio and circularity compared to the parental line. While differences in protein expression (e.g., in the RAS/ERK pathway) have been reported between MDA-BR and MDA-P cell clones (Stark et al., 2007; Dun et al., 2015), here we found only minor differences in shape-based parameters between the MDA-BR and MDA-P clones. Hence, differences in gene and protein expression may not manifest themselves in physical characteristics such as cell morphology on 2D glass or polyacrylamide gels substrates. Interestingly, in our previous work, there were more significant differences in morphology and migration parameters between MDA-P and MDA-BR cells on 2D hyaluronic acid hydrogels (though trends as a function of crosslinking density were similar between clones; Pranda et al., 2019), suggesting that microenvironment parameters are important considerations when comparing across the organotropic clones. Additionally, a previous study showed that within a MDA-MB-231 population, a bone tropic profile was more easily identifiable compared to a brain tropic line based on cell adhesion and spreading (Barney et al., 2015).

While our goal was to characterize basic phenotypic adhesion and morphology parameters of the organotropic clones, we recognize that a limitation of our work is the simplicity of the microenvironments used in the assays. Future work comparing MDA-BO, MDA-BR, and MDA-P cell clones should be focused on studying other cell behaviors and in different microenvironments. For example, in addition to 2D assays, 3D invasion assays (e.g., into collagen gels) would provide information about physiologically relevant cell behavior in the context of tumor cell metastasis (Baskaran et al., 2020). Second, we propose use of more complex systems for modeling combinatorial cues in physiologically-relevant ECMs, including matrix stiffness, soluble factors, and ECM-bound factors. For example, one study assessed the effects of different combinations of ECM proteins on lung adenocarcinoma cell adhesion and found that metastasized tumor cells have a unique adhesion response to certain proteins and that genetic clustering differs in comparison with cells from the primary tumor site (Reticker-Flynn et al., 2012). Additionally, our study used transformed cell lines that were serially passaged in mice and conditioned to metastasize to specific microenvironments in mice. These cells likely do not represent the heterogeneous metastatic breast cancer cells in humans. Future work would benefit from the use of primary cell lines from patients with brain or bone metastasis to compare the mechanobiological phenotypes, since tumor heterogeneity has been shown to influence cell morphology and migration (Davis et al., 2019).

Overall, we have shown that bone-seeking breast tumor clones have some distinct morphological and migratory phenotypes that could be beneficial for navigating the bone microenvironment, in comparison with the brain-seeking or parental clones. Meanwhile, in the limited microenvironments tested, brain-seeking clone behavior showed only very minor differences from the other cell types. Future work should aim to design systems that encompass more complex combinations of cues from the brain microenvironment, such as shear stress, tortuosity, and surrounding cell types. Additionally, further exploring the link between these physical characteristics and gene and protein expression differences found in previous studies could help establish a full profile for differentiating triple negative organotropic tumor cells in a heterogeneous tumor cell population. The next steps towards development of a diagnostic method for organotropic breast cancer would first include comparison of our results with cells from a primary tumor and metastatic sites in humans. Since our experiments used conditioned, transformed cell lines, it is unknown whether the organotropic clones are easily identifiable in a primary tumor, and human samples. If they are identifiable in a primary tumor, a second step would be to develop an efficient microfluidic device, with proper sensitivity and specificity, for imaging and analyzing tumor cell samples based on the mechanobiological phenotypes, and with a goal of analyzing the presence of organotropic breast cancer cells in a primary tumor sample. Likely, such a device would require launching a large set of experiments, with multiple types of ECM compositions, substrate mechanics, topographies, and other microenvironment features, along with the ability to simultaneously measure multiple mechanobiological and migratory behaviors. Sophisticated multivariable analysis techniques would also likely be needed to identify clustering of specific mechanobiological phenotypes in the organotropic breast cancer cells. Hence, while our data presented here are quite limited in clinical relevance, we anticipate that the work could inspire future work towards a mechanobiological phenotyping approach to identify, and/or predict organotropic breast cancer.

## Data Availability

The raw data supporting the conclusion of this article will be made available by the authors, without undue reservation.
