# Supplementary material for: Morphological Phenotyping of Organotropic Brain- and Bone-Seeking Triple Negative Metastatic Breast Tumor Cells
Source: Front Cell Dev Biol. 2022 Feb 17;10:790410. doi: 10.3389/fcell.2022.790410 (PMC8891987; doi:10.3389/fcell.2022.790410)

Supplemental Information for

Morphological phenotyping of organotropic brain- and bone-seeking triple negative metastatic breast tumor cells

Ariana Joy L. DeCastro, Marina A. Pranda, Kelsey M. Gray, John Merlo-Coyne, Nathaniel Girma[1], Madelyn Hurwitz, Yuji Zhang, Kimberly M. Stroka[#]

# Corresponding Author:
Kimberly M. Stroka
3110 A. James Clark Hall
Fischell Department of Bioengineering
University of Maryland, College Park
8278 Paint Branch Drive
College Park, MD 20742
TEL: 301-314-1813
FAX: 301-405-9953
EMAIL: kstroka@umd.edu

Supplemental Tables

Table S1: Results of D’Agostino-Pearson normality test (performed using Graphpad) for all figures from the main manuscript. Cell line clones are abbreviated P (MDA-MB-231 parental cells), BR (MDA-MB-231-BR brain-seeking cells), and BO (MDA-MB-231-BO bone-seeking cells). Table S1 spans the following two pages.

Figure 1B (diameter of suspended cells)

D'Agostino & Pearson test	P	BR	BO
K2	124.9	15.51	53.19
P value	<0.0001	0.0004	<0.0001
Passed normality test (alpha=0.05)?	No	No	No
P value summary	****	***	****

Figure 1C (area of suspended cells)

D'Agostino & Pearson test	P	BR	BO
K2	214.2	25.86	60.02
P value	<0.0001	<0.0001	<0.0001
Passed normality test (alpha=0.05)?	No	No	No
P value summary	****	****	****

Figure 2B (area)

	Passage 6			Passage 7			Passage 10			Passage 12		
D'Agostino & Pearson test	P	BR	BO	P	BR	BO	P	BR	BO	P	BR	BO
K2	198.8	42.64	71.53	119.9	171.7	138.4	82.46	69.45	99.01	111.5	73.53	107.9
P value	<0.0001	<0.0001	<0.0001	<0.0001	<0.0001	<0.0001	<0.0001	<0.0001	<0.0001	<0.0001	<0.0001	<0.0001
Passed normality test (alpha=0.05)?	No	No	No	No	No	No	No	No	No	No	No	No
P value summary	****	****	****	****	****	****	****	****	****	****	****	****

Figure 2C (inverse aspect ratio)

D'Agostino & Pearson test	P	BR	BO	P	BR	BO	P	BR	BO	P	BR	BO
K2	39.39	15.84	19.50	27.59	17.05	16.63	13.94	10.85	13.75	10.67	21.92	9.244
P value	<0.0001	0.0004	<0.0001	<0.0001	0.0002	0.0002	0.0009	0.0044	0.0010	0.0048	<0.0001	0.0098
Passed normality test (alpha=0.05)?	No	No	No	No	No	No	No	No	No	No	No	No
P value summary	****	***	****	****	***	***	***	**	**	**	****	**

Figure 2D (solidity)

D'Agostino & Pearson test	P	BR	BO	P	BR	BO	P	BR	BO	P	BR	BO
K2	21.95	43.33	17.65	5.958	46.00	19.31	15.64	39.15	12.97	9.003	19.73	58.17
P value	<0.0001	<0.0001	0.0001	0.0508	<0.0001	<0.0001	0.0004	<0.0001	0.0015	0.0111	<0.0001	<0.0001
Passed normality test (alpha=0.05)?	No	No	No	Yes	No	No	No	No	No	No	No	No
P value summary	****	****	***	ns	****	****	***	****	**	*	****	****

Figure 2E (circularity)

D'Agostino & Pearson test	P	BR	BO	P	BR	BO	P	BR	BO	P	BR	BO
K2	12.46	2.078	18.74	2.030	10.71	16.67	1.319	2.001	9.496	4.612	4.937	2.703
P value	0.0020	0.3538	<0.0001	0.3624	0.0047	0.0002	0.5170	0.3677	0.0087	0.0996	0.0847	0.2589
Passed normality test (alpha=0.05)?	No	Yes	No	Yes	No	No	Yes	Yes	No	Yes	Yes	Yes
P value summary	**	ns	****	ns	**	***	ns	ns	**	ns	ns	ns

Figure 3B (2D migration speed)

	Collagen I			Fibronectin			Poly – D – Lysine		
D'Agostino & Pearson test	P	BR	BO	P	BR	BO	P	BR	BO
K2	4.153	15.75	10.92	18.24	0.9394	13.72	18.55	8.190	5.951
P value	0.1254	0.0004	0.0043	0.0001	0.6252	0.0010	<0.0001	0.0167	0.0510
Passed normality test (alpha=0.05)?	Yes	No	No	No	Yes	No	No	No	Yes
P value summary	ns	***	**	***	ns	**	****	*	ns

Figure 4B-D (chemotactic index in microchannels)

	P					BR					BO				
Channel Width (μm)	3	6	10	20	50	3	6	10	20	50	3	6	10	20	50
D'Agostino & Pearson test															
K2	4.760	14.86	17.16	44.00	22.54	4.801	44.95	81.83	69.46	24.86	3.721	48.26	62.83	144.4	29.70
P value	0.0925	0.0006	0.0002	<0.0001	<0.0001	0.0907	<0.0001	<0.0001	<0.0001	<0.0001	0.1556	<0.0001	<0.0001	<0.0001	<0.0001
Passed normality test (alpha=0.05)?	Yes	No	No	No	No	Yes	No	No	No	No	Yes	No	No	No	No
P value summary	ns	***	***	****	****	ns	****	****	****	****	ns	****	****	****	****

Figure 4E-G (migration speed in microchannels)

Channel Width (μm)	3	6	10	20	50	3	6	10	20	50	3	6	10	20	50
D'Agostino & Pearson test															
K2	0.9259	70.51	6.088	22.40	1.408	9.826	21.22	4.760	9.933	240.2	2.904	57.88	7.280	95.57	26.76
P value	0.6294	<0.0001	0.0476	<0.0001	0.4945	0.0074	<0.0001	0.0925	0.0070	<0.0001	0.2341	<0.0001	0.0263	<0.0001	<0.0001
Passed normality test (alpha=0.05)?	Yes	No	No	No	Yes	No	No	Yes	No	No	Yes	No	No	No	No
P value summary	ns	****	*	****	ns	**	****	ns	**	****	ns	****	*	****	****

Figure 5B (Young's modulus)

	Glass			194 kPa			1 kPa		
D'Agostino & Pearson test	P	BR	BO	P	BR	BO	P	BR	BO
K2	5.679	4.957	0.4733	14.20	41.94	7.013	50.18	12.10	10.19
P value	0.0585	0.0839	0.7893	0.0008	<0.0001	0.0300	<0.0001	0.0024	0.0061
Passed normality test (alpha=0.05)?	Yes	Yes	Yes	No	No	No	No	No	No
P value summary	ns	ns	ns	***	****	*	****	**	**

Figure 5C (area)

	Glass			194 kPa			1 kPa		
D'Agostino & Pearson test	P	BR	BO	P	BR	BO	P	BR	BO
K2	513.1	371.5	1041	207.6	228.1	94.42	178.0	303.5	143.5
P value	<0.0001	<0.0001	<0.0001	<0.0001	<0.0001	<0.0001	<0.0001	<0.0001	<0.0001
Passed normality test (alpha=0.05)?	No	No	No	No	No	No	No	No	No
P value summary	****	****	****	****	****	****	****	****	****

Figure 5D (inverse aspect ratio)

D'Agostino & Pearson test	P	BR	BO	P	BR	BO	P	BR	BO
K2	102.3	124.4	204.1	5800	4763	269.3	102.1	83.87	118.7
P value	<0.0001	<0.0001	<0.0001	<0.0001	<0.0001	<0.0001	<0.0001	<0.0001	<0.0001
Passed normality test (alpha=0.05)?	No	No	No	No	No	No	No	No	No
P value summary	****	****	****	****	****	****	****	****	****

Figure 5E (circularity)

D'Agostino & Pearson test	P	BR	BO	P	BR	BO	P	BR	BO
K2	22.55	34.82	176.3	1189	4346	81.54	157.2	125.4	3261
P value	<0.0001	<0.0001	<0.0001	<0.0001	<0.0001	<0.0001	<0.0001	<0.0001	<0.0001
Passed normality test (alpha=0.05)?	No	No	No	No	No	No	No	No	No
P value summary	****	****	****	****	****	****	****	****	****

Figure 5F (solidity)

D'Agostino & Pearson test	P	BR	BO	P	BR	BO	P	BR	BO
K2	370.8	362.1	46.23	158.3	92.04	23.52	384.3	440.3	59.37
P value	<0.0001	<0.0001	<0.0001	<0.0001	<0.0001	<0.0001	<0.0001	<0.0001	<0.0001
Passed normality test (alpha=0.05)?	No	No	No	No	No	No	No	No	No
P value summary	****	****	****	****	****	****	****	****	****

Figure 6C (focal adhesion parameters)

PY-paxillin – area

D'Agostino & Pearson test	P	BR	BO
K2	107.5	242.0	393.3
P value	<0.0001	<0.0001	<0.0001
Passed normality test (alpha=0.05)?	No	No	No
P value summary	****	****	****

Phospho-FAK - area

D'Agostino & Pearson test	P	BR	BO
K2	333.0	257.2	292.3
P value	<0.0001	<0.0001	<0.0001
Passed normality test (alpha=0.05)?	No	No	No
P value summary	****	****	****

of PY-paxillin per cell

D'Agostino & Pearson test	P	BR	BO
K2	9.006	23.03	45.42
P value	0.0111	<0.0001	<0.0001
Passed normality test (alpha=0.05)?	No	No	No
P value summary	*	****	****

of Phospho-FAK per cell

D'Agostino & Pearson test	P	BR	BO
K2	4.865	9.194	14.76
P value	0.0878	0.0101	0.0006
Passed normality test (alpha=0.05)?	Yes	No	No
P value summary	ns	*	***

PY-paxillin density

D'Agostino & Pearson test	P	BR	BO
K2	3.324	7.856	12.31
P value	0.1898	0.0197	0.0021
Passed normality test (alpha=0.05)?	Yes	No	No
P value summary	ns	*	**

Phospho-FAK density

D'Agostino & Pearson test	P	BR	BO
K2	6.255	43.20	17.74
P value	0.0438	<0.0001	0.0001
Passed normality test (alpha=0.05)?	No	No	No
P value summary	*	****	***

Table S2: Statistical comparisons for morphological parameters of MDA-P, MDA-BR, and MDA-BO cells on collagen I-coated polyacrylamide gels of varying stiffness (corresponds to Figure 5). * indicates P<0.05. ** indicates P<0.01. *** indicates P<0.001. **** indicates P<0.0001.

Area							
Comparisons	**Glass**	**194 kPa**	**1 kPa**	Comparisons	**MDA-P**	**MDA-BR**	**MDA-BO**
P vs BR	**	**	ns	Glass vs 194 kPa	****	****	****
P vs BO	****	****	****	Glass vs 1 kPa	****	****	****
BR vs BO	****	****	****	194 kPa vs 1 kPa	****	****	****

Inverse Aspect Ratio							
Comparisons	**Glass**	**194 kPa**	**1 kPa**	Comparisons	**MDA-P**	**MDA-BR**	**MDA-BO**
P vs BR	**	ns	***	Glass vs 194 kPa	****	****	ns
P vs BO	**	ns	****	Glass vs 1 kPa	****	****	****
BR vs BO	****	ns	**	194 kPa vs 1 kPa	****	****	****

Solidity							
Comparisons	**Glass**	**194 kPa**	**1 kPa**	Comparisons	**MDA-P**	**MDA-BR**	**MDA-BO**
P vs BR	**	*	****	Glass vs 194 kPa	**	ns	****
P vs BO	****	****	****	Glass vs 1 kPa	****	****	****
BR vs BO	****	****	****	194 kPa vs 1 kPa	****	****	****

Circularity							
Comparisons	**Glass**	**194 kPa**	**1 kPa**	Comparisons	**MDA-P**	**MDA-BR**	**MDA-BO**
P vs BR	**	ns	**	Glass vs 194 kPa	***	***	**
P vs BO	****	****	****	Glass vs 1 kPa	****	****	****
BR vs BO	****	****	****	194 kPa vs 1 kPa	****	****	****

Cell line authentication

Upon arriving in our lab, the cells obtained from Dr. Toshiyuki Yoneda in Osaka, Japan were checked for authenticity using STR testing via Laragen, Inc. Results from the cell line authentication for the three cell lines, as provided by Laragen, Inc., are included on the following pages.

Sample 1 = MDA-MB-231 parental (MDA-P)

Sample 2 = MDA-MB-231 brain-seeking (MDA-BR)

Sample 3 = MDA-MB-231 bone-seeking (MDA-BO)

Report Date: May 9th, 2018

Celient: Marina Shumakovich | PI: Dr. Stroka
Institution: U of Maryland | PO: CC
Phone: 301-314-1813 | Email: mshumako@terpmail.umd.edu
Address: 8278 Paint Branch Dr. 3107A james clark Hall

Summary of the Report: Authentication for all cell lines used in this study was performed using the Promega powerplex16 System recommended by American Type Culture Collection. The STR alleles were searched either on ATTC or DSMZ databases depending availability of the cell lines in the databases.

Sample ID: 1

Sample DSMZ#: HTB-26

DSMZ Database Best Match Score: 0.89

DSMZ Database Best Match Cell Line: MDA-MB-231

Marker	Dye	Allele 1	Allele 2	Allele 3	DSMZ
D5S818	G	12			12,12
D13S317	G	13			13,13
D7S820	G	8			8,9
D16S539	G	12			12,12
vWA	Y	15			15,18
TH01	B	7	9.3		7,9.3
AMEL	Y	X			X,X
TPOX	Y	8	9		8,9
CSF1PO	G	12	13		12,13
D18S51	B	11	16		
D21S11	B	30	33.2		
D3S1358	B	16			
D8S1179	Y	13			
FGA	Y	22	23		
Penta_D	G	11	14		
Penta_E	B	11			

Applied Biosystems
GeneMapper Software 5

051018-cellid

Sample File	Panel	OS	SQ
1-12-1 D03.fsa	PowerPlex 16 ID3.2.0		

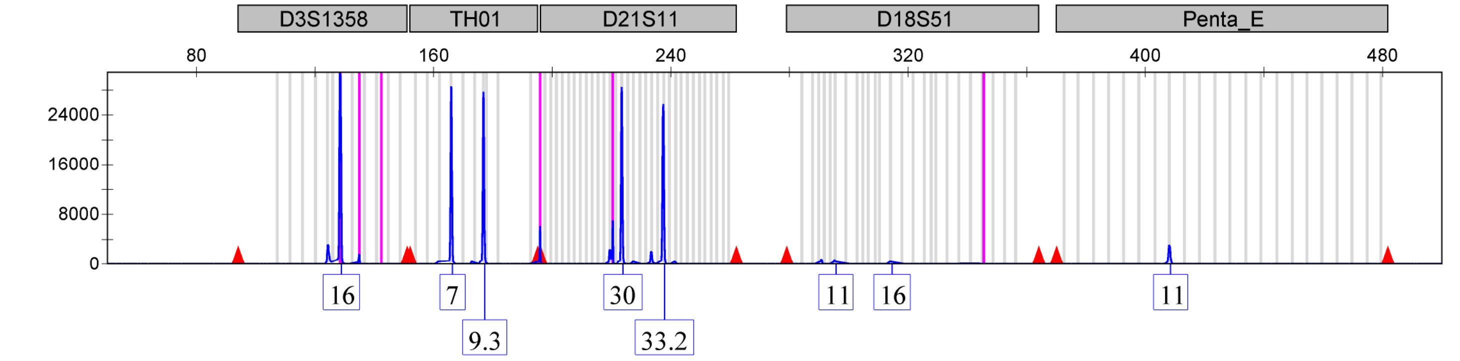

1-12-1 D03.fsa	PowerPlex 16 ID3.2.0		

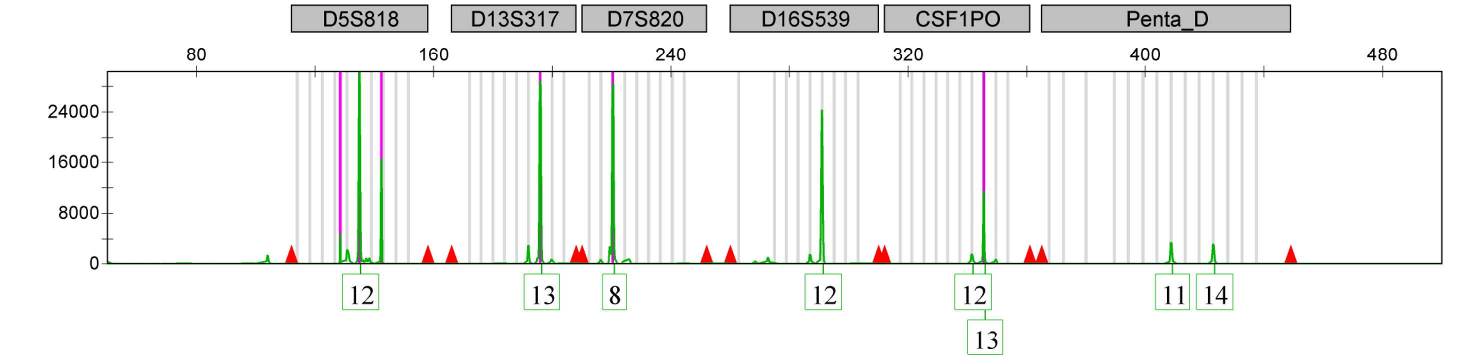

1-12-1 D03.fsa	PowerPlex 16 ID3.2.0		

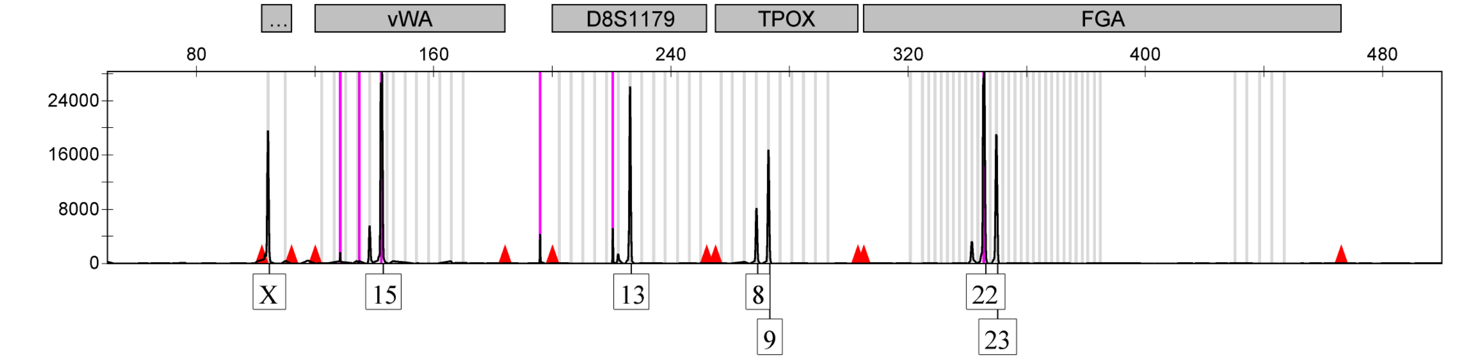

Sample File	Panel	OS	SQ
1-12-1_D03.fsa	PowerPlex_16_ID3.2.0		

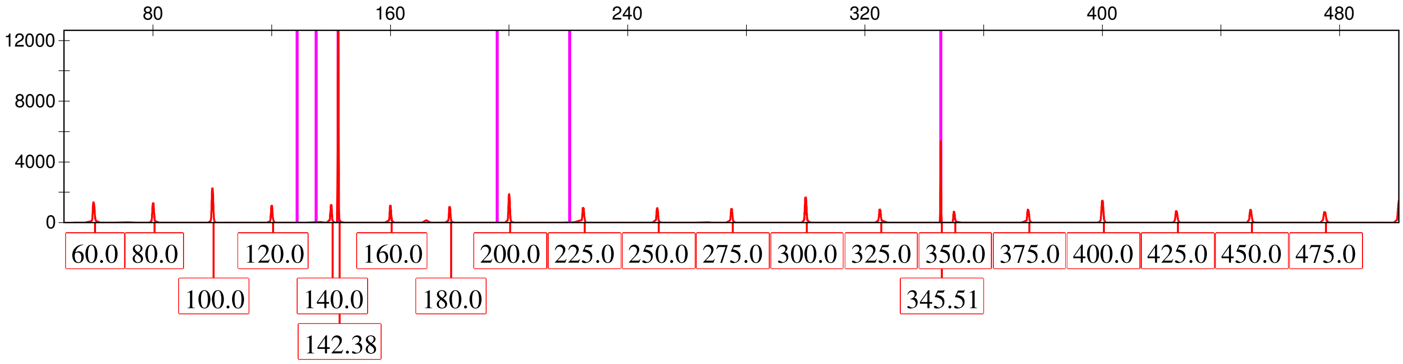

Sample ID: 2

Sample DSMZ#: HTB-26

DSMZ Database Best Match Score: 0.89

DSMZ Database Best Match Cell Line: MDA-MB-231

Marker	Dye	Allele 1	Allele 2	Allele 3	DSMZ
D5S818	G	12			12,12
D13S317	G	13			13,13
D7S820	G	8			8,9
D16S539	G	12			12,12
vWA	Y	15			15,18
TH01	B	7	9.3		7,9.3
AMEL	Y	X			X,X
TPOX	Y	8	9		8,9
CSF1PO	G	12	13		12,13
D18S51	B	11	16		
D21S11	B	30	33.2		
D3S1358	B	16			
D8S1179	Y	13			
FGA	Y	22	23		
Penta_D	G	11	14		
Penta_E	B	11			

Applied Biosystems

GeneMapper Software 5

051018-cellid

Sample File	Panel	OS	SQ
1-13-2_E03.fsa	PowerPlex_16_ID3.2.0		

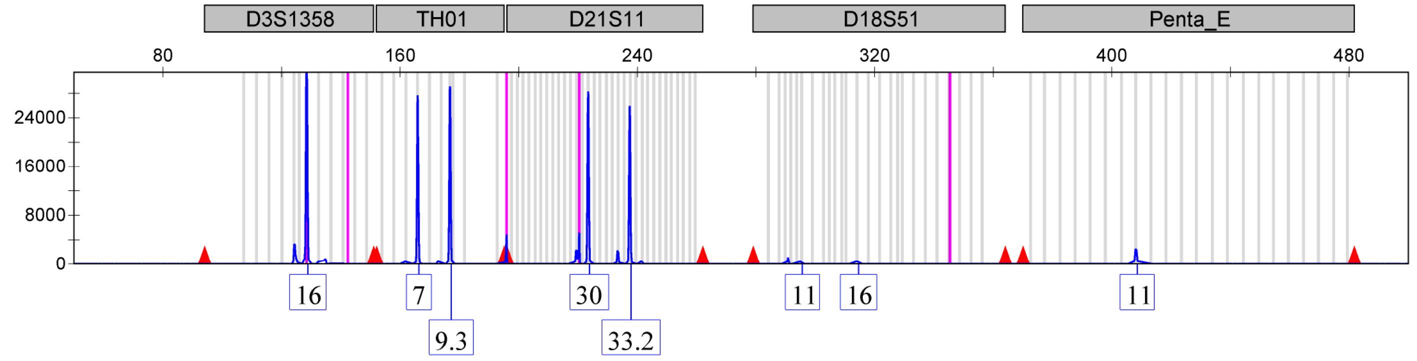

1-13-2_E03.fsa	PowerPlex_16_ID3.2.0		

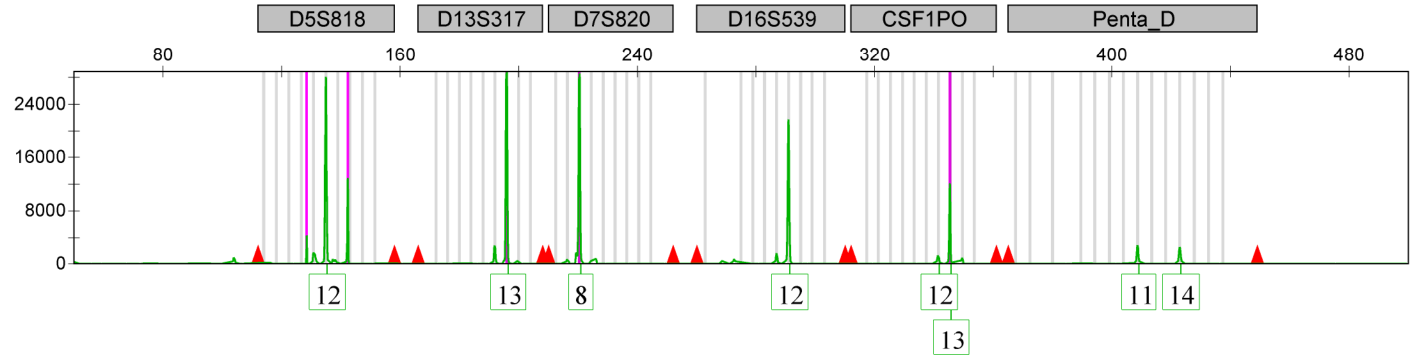

1-13-2_E03.fsa	PowerPlex_16_ID3.2.0		

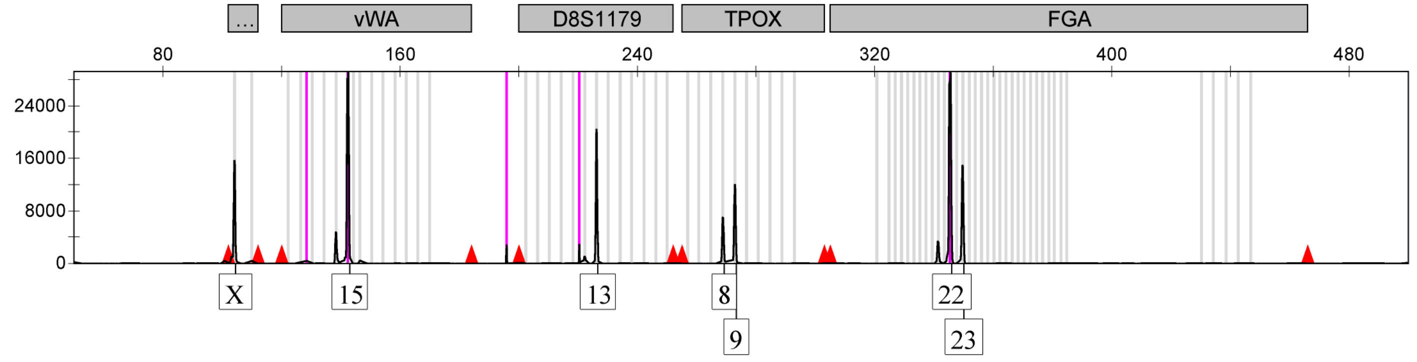

Sample File	Panel	OS	SQ
1-13-2_E03.fsa	PowerPlex_16_ID3.2.0		

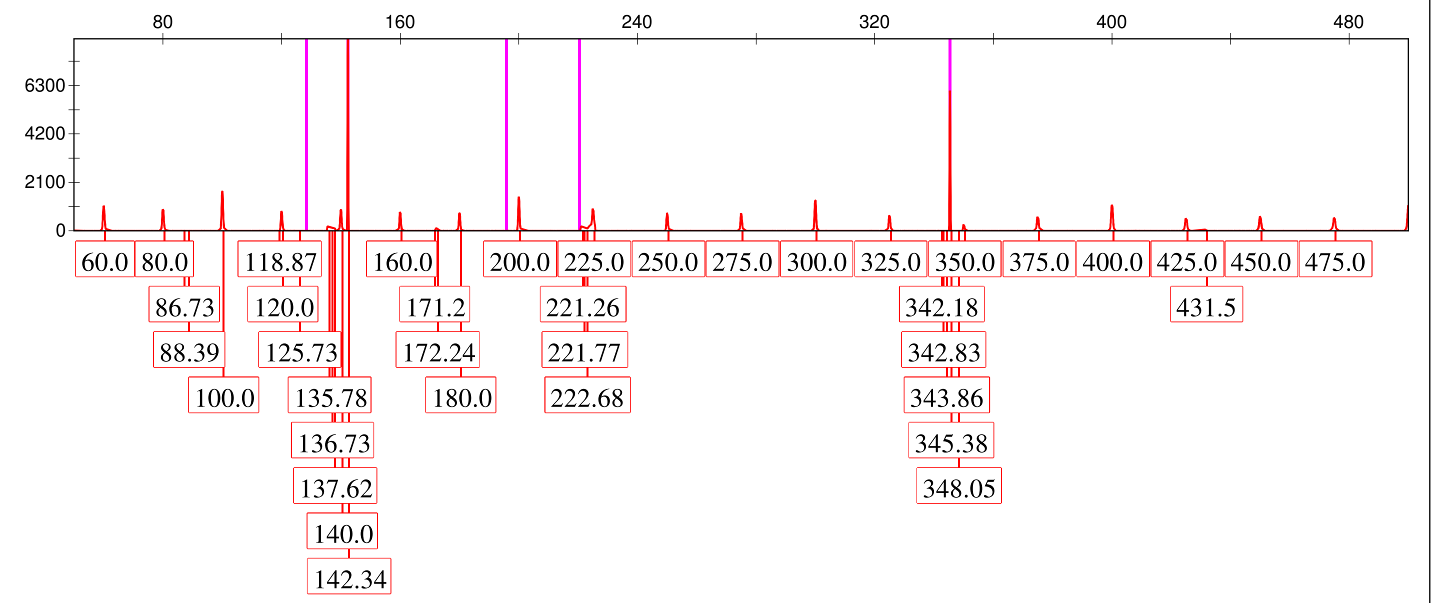

Sample ID: 3

Sample DSMZ#: HTB-26

DSMZ Database Best Match Score: 0.89

DSMZ Database Best Match Cell Line: MDA-MB-231

Marker	Dye	Allele 1	Allele 2	Allele 3	DSMZ
D5S818	G	12			12,12
D13S317	G	13			13,13
D7S820	G	8			8,9
D16S539	G	12			12,12
vWA	Y	15	16		15,18
TH01	B	7	9.3		7,9.3
AMEL	Y	X			X,X
TPOX	Y	8	9		8,9
CSF1PO	G	12	13		12,13
D18S51	B	11	16		
D21S11	B	30	33.2		
D3S1358	B	16			
D8S1179	Y	13			
FGA	Y	22	23		
Penta_D	G	11	14		
Penta_E	B	11			

Sample File	Panel	OS	SQ
1-14-3 F03.fsa	PowerPlex 16 ID3.2.0		

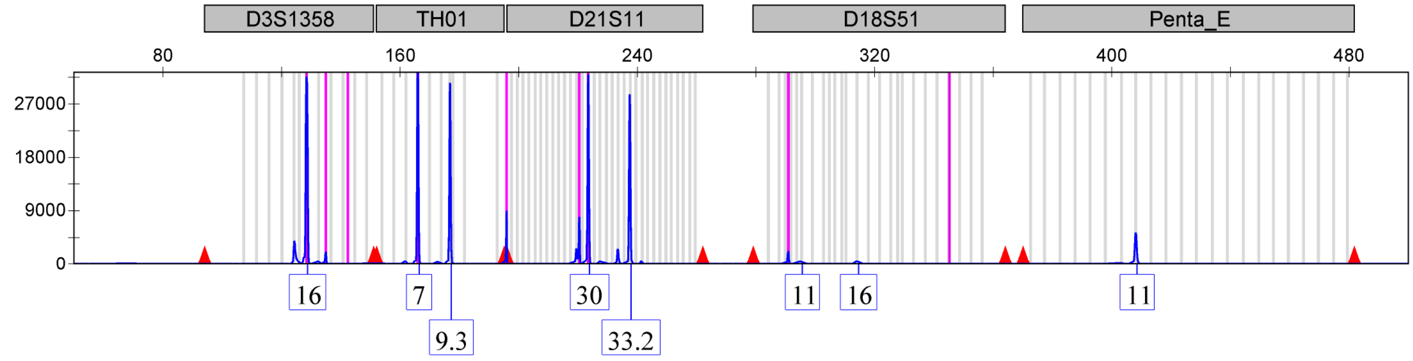

Sample File	Panel	OS	SQ
1-14-3 F03.fsa	PowerPlex 16 ID3.2.0		

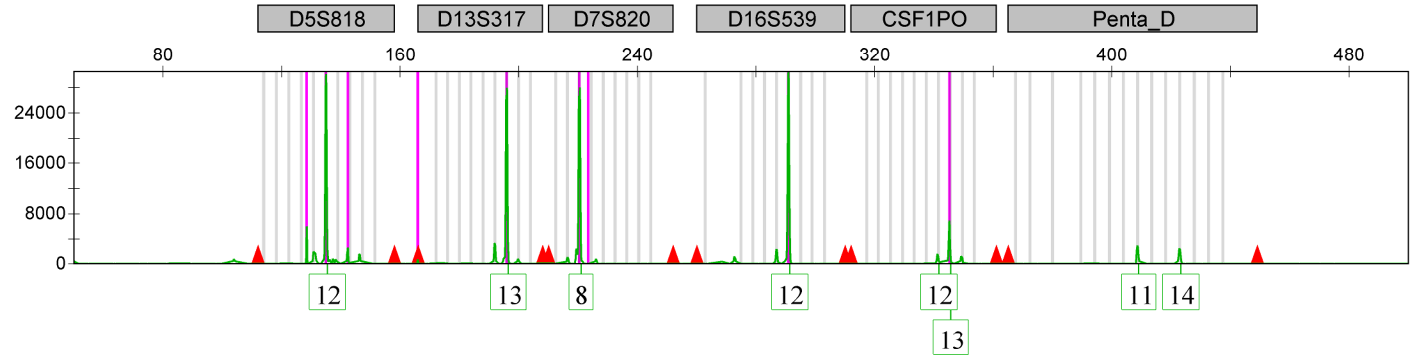

Sample File	Panel	OS	SQ
1-14-3 F03.fsa	PowerPlex 16 ID3.2.0		

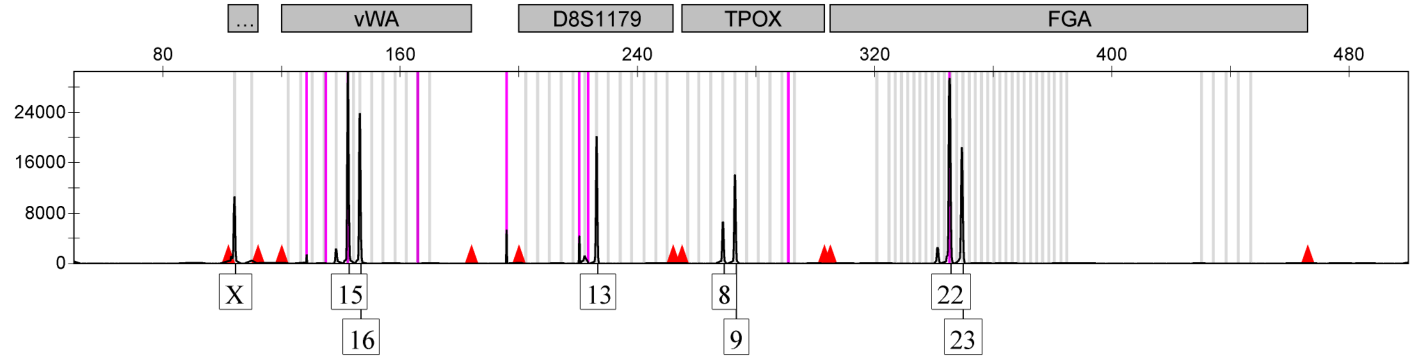

Applied Biosystems
GeneMapper Software 5

051018-cellid

Sample File	Panel	OS	SQ
1-14-3_F03.fsa	PowerPlex_16_ID3.2.0		

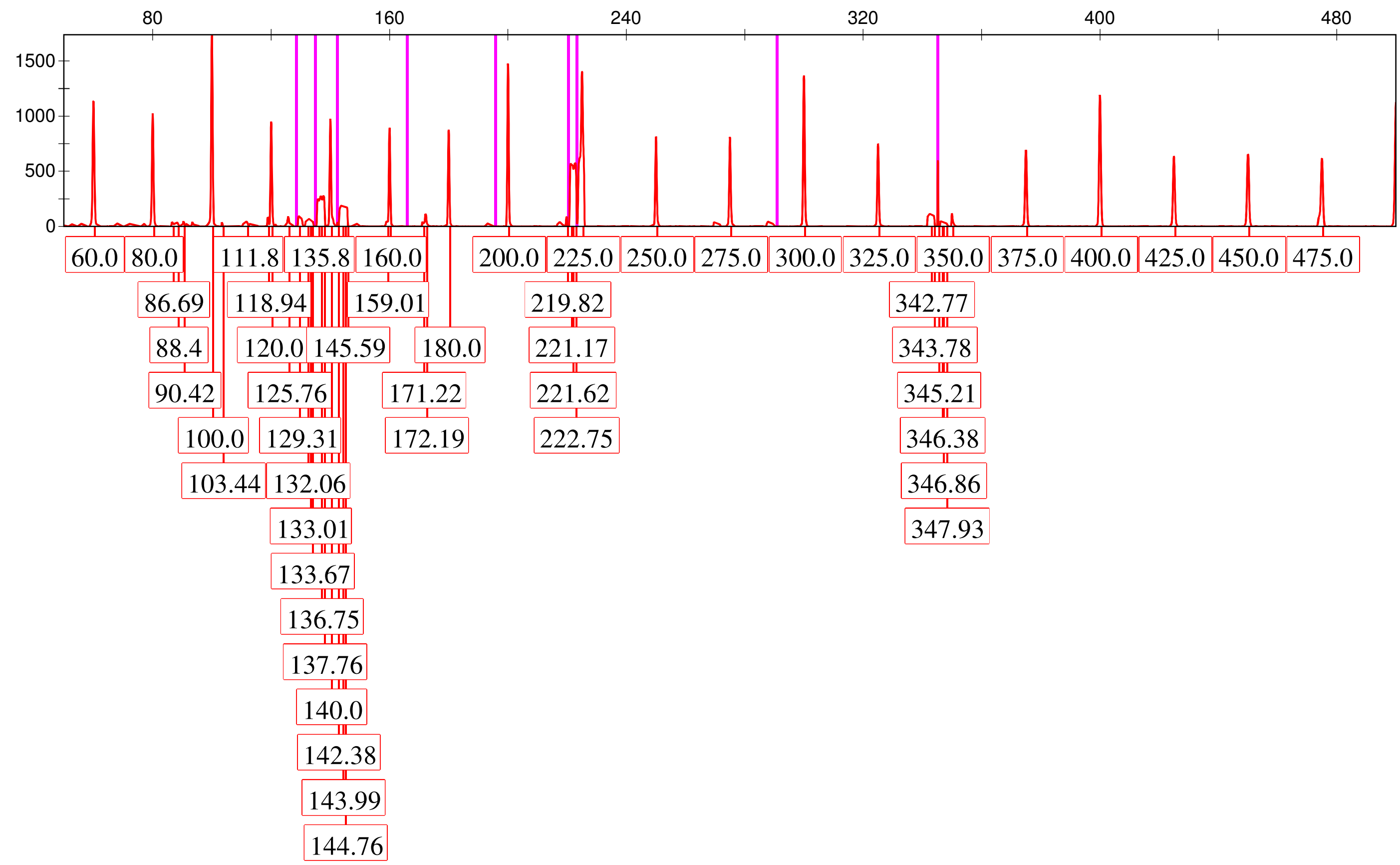

Supplement: Supplementary file 1 [file DataSheet1.PDF]
